# Evaluation of Autonomic Nervous System Dysfunction in Childhood Obesity and Prader–Willi Syndrome

**DOI:** 10.3390/ijms24098013

**Published:** 2023-04-28

**Authors:** Lawrence P. Richer, Qiming Tan, Merlin G. Butler, Hayford M. Avedzi, Darren S. DeLorey, Ye Peng, Hein M. Tun, Arya M. Sharma, Steven Ainsley, Camila E. Orsso, Lucila Triador, Michael Freemark, Andrea M. Haqq

**Affiliations:** 1 Department of Pediatrics, University of Alberta, Edmonton, AB T6G 2R3, Canada; 2 Departments of Psychiatry & Behavioral Sciences and Pediatrics, Kansas University Medical Center, Kansas City, KS 66160, USA; 3 Faculty of Kinesiology, Sport, and Recreation, University of Alberta, Edmonton, AB T6G 2R3, Canada; 4 JC School of Public Health, Faculty of Medicine, The Chinese University of Hong Kong, Hong Kong 999077, China; 5 Department of Medicine, University of Alberta, Edmonton, AB T6G 2R3, Canada; 6 Department of Agricultural Food & Nutritional Science, University of Alberta, Edmonton, AB T6G 2R3, Canada; 7 Division of Pediatric Endocrinology, Duke University Medical Center, Durham, NC 27705, USA

**Keywords:** autonomic nervous system (ANS), childhood obesity, genetics, Prader–Willi syndrome (PWS)

## Abstract

The autonomic nervous system (ANS) may play a role in the distribution of body fat and the development of obesity and its complications. Features of individuals with Prader–Willi syndrome (PWS) impacted by PWS molecular genetic classes suggest alterations in ANS function; however, these have been rarely studied and presented with conflicting results. The aim of this study was to investigate if the ANS function is altered in PWS. In this case-control study, we assessed ANS function in 20 subjects with PWS (6 males/14 females; median age 10.5 years) and 27 body mass index (BMI) z-score-matched controls (19 males/8 females; median age 12.8 years). Standardized non-invasive measures of cardiac baroreflex function, heart rate, blood pressure, heart rate variability, quantitative sudomotor axon reflex tests, and a symptom questionnaire were completed. The increase in heart rate in response to head-up tilt testing was blunted (*p* < 0.01) in PWS compared to controls. Besides a lower heart rate ratio with Valsalva in PWS (*p* < 0.01), no significant differences were observed in other measures of cardiac function or sweat production. Findings suggest possible altered sympathetic function in PWS.

## 1. Introduction

Childhood obesity, regardless of the cause, increases risks for early onset cardio-metabolic morbidity, premature mortality, and higher healthcare costs [1,2,3]. Prader–Willi syndrome (PWS) is the most common syndromic form of obesity and recognized as an extreme model in humans. PWS is a multisystem genetic disorder that occurs in all races and ethnicities with comparable rates in males and females [4,5]. PWS is due to errors in genomic imprinting, most often by a paternal loss of expression of imprinted genes in the 15q11-q13 region [6,7,8].

Approximately 60% of individuals with PWS show a paternally derived 15q11-q13 deletion (DEL15), 35% have uniparental disomy 15 (UDP15), or both 15s from the mother; while the remaining have imprinting center defects, translocations or inversions involving chromosome 15 [8]. Clinical and behavioral differences have been identified in those with PWS having the 15q11-q13 deletion or with maternal disomy 15. Those with maternal disomy 15 have a higher verbal IQ, delayed diagnosis, and more psychosis or autistic behavior than those with the deletion [9,10,11]. The individuals with PWS and the deletion are more prone to have compulsions, self-injury, higher pain threshold and hypopigmentation compared with those having maternal disomy 15 [9,12,13,14,15,16].

**Figure 1:**
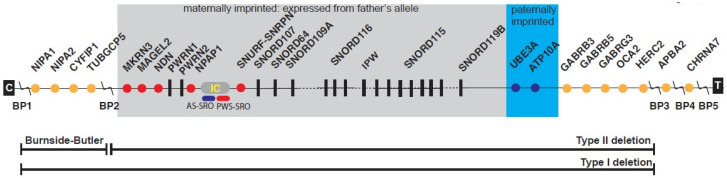
Linear order of genes and transcripts with their location from the proximal long arm of chromosome 15 in Prader–Willi syndrome (PWS). Three recognized deletions are shown including the larger typical 15q11-q13 Type I deletion involving breakpoints BP1 and BP3, and smaller typical 15q11-q13 Type II deletion involving BP2 and BP3, both causing PWS and Angelman syndrome (AS) depending on the parent of origin. The 15q11.2 BP1-BP2 deletion (Burnside–Butler) involves only BP1 and BP2. Nonimprinted genes are shown in orange while maternally imprinted genes (paternally expressed) are shown in red and paternally imprinted genes (maternally expressed) are shaded in blue. Abbreviations: BP, breakpoint; IC, imprinting center; PWS-SRO, PWS smallest region of overlap; AS-SRO, AS smallest region of overlap; C and T, centromere and telomere locations (from Butler, Lee, and Whitman [9] with permission from publisher).

Dozens of genes and/or transcripts are identified between chromosome 15q11-q13 breakpoints associated with PWS and are prone to non-homologous recombination leading to 15q11-q13 deletions observed in PWS. Genes in the 15q11-q13 region contain both imprinted (NDN, MAGEL2, MKRN3, SNURF-SNRPN, SNORDs, UBE3A, ATP10A) and non-imprinted (NIPA1, NIPA2, CYFIP1, TUBGCP5, GABA receptors, OCA2) genes [6,7,8]. These genes and their encoded protein functions are implicated in neurodevelopment, motor control, behavioral issues, learning disabilities, autism, hyperphagia, obesity, hypogonadism, infertility, precocious puberty, sleep disturbances, skin pigment production, and hormone and metabolic disturbances (Figure 1) [6,7,8,9,16,17].

The autonomic nervous system (ANS) involvement in the pathogenesis of obesity and PWS has been summarized previously [18,19]. The ANS, composed of the central autonomic network and peripheral components (i.e., sympathetic, parasympathetic, and enteric nervous systems), maintains homeostasis by regulating essential physiological processes such as heart rate, blood pressure, breathing, digestion, body temperature, hormone production, and sexual arousal with many of these features disturbed in PWS [20]. Individuals with PWS often exhibit oropharyngeal and bowel dysmotility, abnormal temperature regulation (reduction in core temperature in response to cold stress), altered sleep control (excessive daytime somnolence, a primary abnormality of the circadian rhythm of rapid eye movement sleep, and insensitivity to hypoxia and hypercarbia), altered perception of pain (markedly altered cold and heat pain sensations not attributed to peripheral nerve involvement) [21], and decreased salivation [22,23]. Functional magnetic resonance imaging (MRI) studies of adults with PWS have shown significant delay in activation of areas subserving satiety and autonomic function including the hypothalamus, insula, ventromedial prefrontal cortex, and nucleus accumbens [24]. Other investigations have shown alternation in the medial prefrontal cortex (MPFC)/inferior parietal lobe (IPL), MPFC/precuneus, IPL/precuneus, and IPL/hippocampus, and other regions in the resting state.

Prefrontal circuitry is an important factor determining hunger and satiety. When compared with simple obesity and healthy weight controls, those with PWS demonstrated higher activity in the reward/limbic regions (nucleus accumbens, amygdala) and lower activity in the hypothalamus and hippocampus in response to food versus non-food images before food consumption [25,26,27,28,29]. In PWS, hyperactivations in subcortical reward circuitry and hypoactivations in cortical inhibitory regions were found after eating which provided evidence of neural substrates associated with variable abnormal food motivation phenotypes in PWS and those with simple obesity. The results point to distinct neural mechanisms associated with hyperphagia in PWS. These studies lend evidence for the role of the ANS in obesity and specifically in PWS. PWS is recognized as an extreme form of obesity in humans and can serve as a unique model to study the functional connectivity in brain regions implicated in eating, reward, and ANS function with alterations in PWS [30].

Startle response as an involuntary reflex activated by the ANS has been investigated in PWS [31]. This reflex is in response to sudden or disturbed auditory/visual stimuli which may be modulated by the emotional valence of concurrently viewed visual stimuli. The subjective ratings of food images and urge to eat were significantly higher in PWS than controls and did not significantly decline post meal. Acoustic startle response was detected in PWS but was significantly lower than controls under all conditions. Abnormal responses to food images in PWS were attenuated relative to other picture types with potentially abnormal emotional modulation of responses to non-food images which contrasted self-reported picture ratings. A stable positive emotional valence to food images was observed pre-and post-feeding with a sustained urge to consume food in PWS. Possibly, a disruption of the autonomic or sympathetic nervous system functioning reported in PWS may impact on hunger and/or food drive states. These studies support the feasibility of eyeblink startle modulation to assess food motivation in PWS and provide preliminary data to stimulate additional studies. These studies would optimize methodological parameters to judge more objectively hyperphagia in PWS than currently available subjective hyperphagia questionnaire forms in use.

Experimental animal models of PWS suggest specific abnormalities in the development of the sympathetic nervous system [32]. Necdin is one of several proteins that are genetically inactivated in PWS and it is important in the differentiation of sensory neurons. Necdin-null mice have diminished formation, migration, and survival of the sympathetic superior cervical ganglion neurons (the most rostral of the paravertebral sympathetic ganglions innervating the pupil, lacrimal, salivary glands, and cerebral blood vessels) and reduced innervation of target systems [32]. Furthermore, axonal extension is impaired throughout the sympathetic nervous system in necdin-null mice.

Unlike exogenous childhood obesity, which is caused primarily by external modifiable risk factors such as physical inactivity and unhealthy diet, PWS is characterized by certain hormonal and biochemical adaptations that may increase the risk for obesity [19]. For example, adolescents and children with PWS have higher insulin sensitivity than children with comparable body mass index (BMI) z-scores [33] and have higher fasting and post-prandial levels of ghrelin, a circulatory peptide produced in the stomach that stimulates appetite and promotes weight gain [34,35,36]. Likewise, the ratio between acylated and unacylated ghrelin was elevated in a recent study of 138 children and adults with PWS [37].

Food intake is controlled by the hypothalamus including the melanocortin and neuropeptide Y (NPY) systems in the arcuate nucleus [34,35]. Ghrelin is short-lived but stimulates eating behavior while peptide YY (PYY), released post-prandially in proportion to caloric intake, remains elevated for several hours and decreases eating [36,38]. Ghrelin levels are inversely correlated with body weight, higher during starvation and increased during weight loss in humans [36,38]. Total ghrelin levels are suppressed in non-PWS children and adults with exogenous obesity or with obesity caused by mutations in leptin or the melanocortin-4 receptor [39]. The mechanisms underlying hyperghrelinemia in PWS remain unclear at this time but prohormone convertase PC1 required to cleave inactive prohormones including POMC (pro-opio-melanocortin, key for hypothalamic appetite regulation) into individual active peptides appears abnormal in PWS. It may play a role in hormone production and function in PWS [40]. Yet, two recent open trials of vagus nerve stimulation showed improvements in maladaptive behavior, temperament, social functioning, and food-seeking behavior, but not weight in adults with PWS [41,42]. These findings suggest that increased vagal nerve efferent activity to the gastrointestinal tract in PWS may contribute to the higher concentrations of ghrelin seen in this disorder. Furthermore, gene expression studies of ghrelin detected no differences in the pattern of gene expression in the brain between those with or without PWS [43].

Recent studies have shown that prohormone convertase PC1 disturbances are associated with the PWS clinical phenotype impacting several hormones in PWS. These hormone disturbances lead to hyperphagic obesity, short stature, hypogonadism, growth and other hormone deficiencies, high ghrelin, and relatively low insulin levels thought to be due to impaired prohormone processing [40]. Burnett et al. [40] reported reduced levels and activity of prohormone convertase PC1 in PWS and proposed that humans and mice deficient in PC1 display hyperphagic-related obesity. Many PWS features may be due to impaired prohormone processing and lack of active hormones required for normal multiple organ systems in PWS. For example, POMC is a large prohormone that requires cleavage from an inactive prohormone status to smaller active peptides for normal appetite regulation and eating behavior. Several major abnormal neuroendocrine clinical findings seen in PWS could be due to PC1 deficiency also impacting ANS function [18,33,44,45].

Few human studies have evaluated autonomic regulation in PWS and have typically used only indirect measures of autonomic function [19]. One study reported diminished parasympathetic nervous system function based on findings of higher resting heart rate and reduced increases in diastolic blood pressure upon standing [46]. However, when controlling for BMI, other studies report no differences in the autonomic control of cardiac reflexes on heart rate and blood pressure in PWS subjects [47]. The purpose of the present study was to determine if autonomic symptoms and metrics of cardiac autonomic reflex control and post-ganglionic autonomic innervation of sweat glands differ between children with PWS and age- and BMI z-score-matched controls.

## 2. Results

### 2.1. Participant Characteristics

Baseline participant characteristics are summarized in Table 1. Twenty children with PWS (median age and BMI z-score: 10.8 years and 0.7, respectively) participated in this study. We recruited 27 control subjects with age and BMI z-scores as close as possible to participants with PWS (median age and BMI z-score: 12.8 years and 0.2, respectively). Some controls were siblings or friends of the participants with PWS, while others were recruited from the local Pediatric Centre for Weight and Health. Due to random chance, a higher percentage of children with PWS were female (70%) as opposed to the control group (30%). Eleven subjects with PWS were taking recombinant human growth hormone (rhGH) (mean dose 0.02 mg/kg/d) at the time of the study [48]. Referring physicians made the initial decisions whether to treat with rhGH and patients had been receiving GH intervention for at least 1 year at the time of study. There was no significant difference in characteristics between PWS patients and PWS patients on rhGH intervention (*p* > 0.05). All participants with PWS had free thyroxine (T4) and thyroid stimulating hormone (TSH) levels in the normal range (either endogenous or on replacement); three participants with PWS were on thyroid replacement to treat central hypothyroidism.

### 2.2. Genetics of PWS Patients

Of the 20 PWS patients, 12 patients had the 15q11-q13 deletion (DEL15) form of the syndrome, 6 patients had uniparental disomy 15 (UPD15), whereas 2 cases were unclear. There was no significant difference between the characteristics for DEL15 patients and UPD15 patients (*p* > 0.05) (Table 2).

### 2.3. Autonomic Symptom Questionnaire

**Figure 2:**
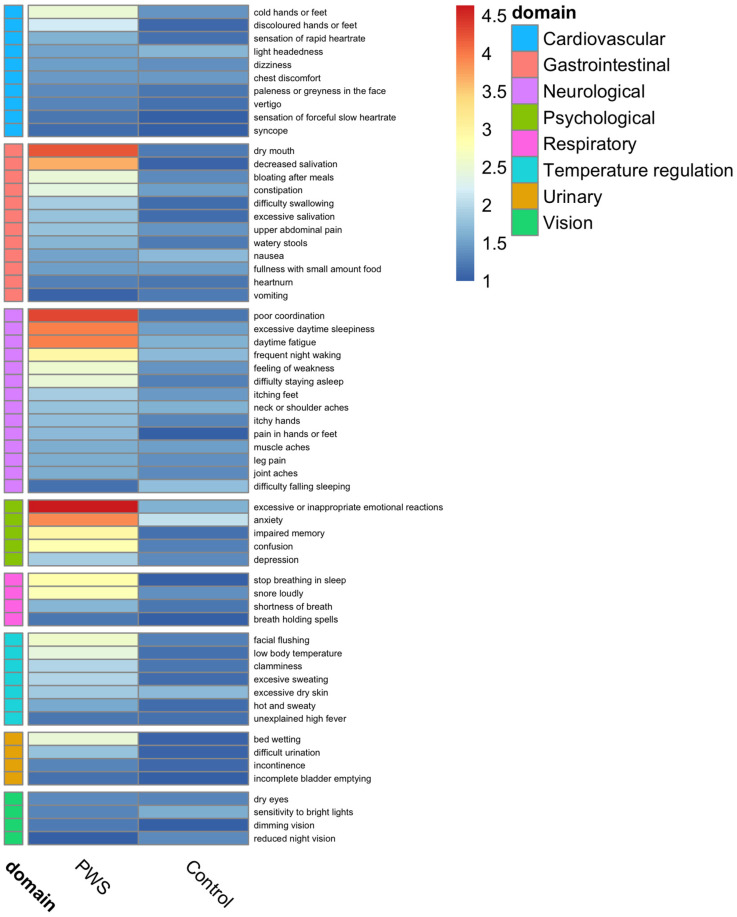
Evaluation of the presence and frequency of cardiovagal, gastrointestinal, thermoregulatory, neuropathic, and psychological symptoms in Prader–Willi Syndrome and control groups. Ordinal frequency scale: 0 = never, 1 = <once per month, 2 = 2–4 times per month, 3 = 5–7 times per month, 4 = most days, and 5 = daily.

An ANS symptom questionnaire was used to describe the self-reported presence and frequency of symptoms on an ordinal scale: 0 = never, 1 = <once per month, 2 = 2–4 times per month, 3 = 5–7 times per month, 4 = most days, and 5 = daily. Of this cohort, 90% (*n* = 18) of the participants with PWS and 70% (*n* = 19) of the controls completed their ANS evaluation. Comparison of results in PWS individuals versus controls is shown in a heatmap (Figure 2). There were significant differences in the presence and frequency of various domains, including cardiovascular (i.e., discoloration of hands or feet), gastrointestinal (i.e., dry mouth, decreased saliva secretion, bloating, feeding difficulty, and food preoccupation), thermoregulation (i.e., flush, low temperature, and excess sweat), neurological (i.e., lack of coordination in movement, fatigue, excessive daytime sleepiness, pain tolerance, sleeping issues, feeling of weakness, and pain in hands/feet), and psychological (excessive/inappropriate emotional reactions, anxiety, socialization, memory, confusion, learning disability, school performance, and tics) symptoms between the two groups. Dry mouth and decreased saliva secretion, lack of coordination in movement, and excessive/inappropriate emotional reactions were particularly common in PWS.

### 2.4. Clinical Autonomic Cardiac Reflex Testing

Results of the clinical autonomic cardiac reflex tests, including 70 degrees head-up tilt (HUT), Valsalva maneuver, and heart rate deep breathing (HRDB), and associated heart rate and non-invasive blood pressure and group comparisons are shown in Table 3. There was a significant difference between PWS and control groups in the maximum change in heart rate from baseline on the HUT test (*p* < 0.01). On Valsalva maneuver, the median heart rate ratio with Valsalva was significantly lower in the PWS group (*p* < 0.01) compared with controls at 1.68 (95% CI 1.45, 1.92). However, the heart rate ratio in the PWS group was above the normal threshold of 1.20 used in our clinical laboratory. In the per protocol analysis, the heart rate ratio with Valsalva was 1.92 (95% CI 1.63, 2.06) in the PWS group, and the difference between groups was no longer significant (*p* = 0.059). The average heart rate difference between inspiration and expiration with HRDB was not significantly different between groups (Table 3).

### 2.5. Heart Rate Variability

Heart rate variability, which provides a secondary measure of sympathetic and parasympathetic tone on heart rate during the short term (5 min) component of HUT and 5 min of supine rest, was not significantly different between groups. Time domain metrics, included RMSSD, NN50, pNN50 [49], were not significantly different between groups at rest or HUT. Frequency domain measures included low frequency (LF)/high frequency (HF) power and were not significantly different between groups at rest (Controls 1.18 (95% CI 0.48, 2.02) and PWS 1.36 (95% CI 0.64, 1.88); *p* = 0.8) but were significantly different with HUT (Controls 1.34 (95% CI 0.99, 2.28) and PWS 2.57 (95% CI 1.52, 4.62); *p* = 0.035).

### 2.6. Quantitative Sudomotor Axon Reflex Test

The volume of quantitative sudomotor axon reflex test (QSART) was not well tolerated among participants with missing data at one or more testing among 19 of 20 in the control group and 23 of 24 in the PWS group. The forearm and proximal leg sites had fewer participants missing with 2 in the control group and 12 in the PWS group. Non-parametric analysis of available data showed no statistically significant difference between groups, but the frequency of missing data and low power makes interpretation problematic. Potential group differences in stroke volume were not measured.

### 2.7. Pupillometry

The pupillometry procedure was also poorly tolerated among participants and many refused the procedure. With available data, the mean increase in pupil size (mydriasis) following the application of 5% cocaine to the conjunctiva was normally distributed and not significantly different (*p* = 0.532) between groups, with a mean of 1.51 mm (SD 0.85, *n* = 9) for the controls and 1.28 mm (SD 0.71, *n* = 10) for participants with PWS. There was, however, a significant difference in the baseline pupil size (*p* = 0.038); the control group members had a larger baseline pupil size (mean 5.21 mm, SD 0.52) than the PWS group members (mean 4.59 mm, SD 0.67).

## 3. Discussion

This study investigated the clinical autonomic phenotype, cardiac autonomic reflexes, and peripheral small nerve fiber sudomotor (sweat) function of 47 participants, including 20 individuals with PWS and 27 BMI z-score-matched controls. The most striking observation was the notable participant and parent-reported differences in the frequency of signs and symptoms often associated with peripheral autonomic nerve dysfunction like dry mouth, decreased salivation, pain insensitivity as well as more general symptoms often associated with autonomic dysfunction like fatigue and daytime sleepiness. The study aimed to examine measures of autonomic function including cardiac reflexes, heart rate variability, sudomotor function, and pupillometry. The primary observation was a blunted heart rate response to HUT among PWS participants compared with age and weight-matched controls. This finding may suggest a blunted sympathetically mediated heart rate response to HUT or higher vagally mediated parasympathetic tone. There were no significant associated differences observed in the decrease of systolic blood pressure with HUT, suggesting peripheral autonomic control, including sympathetic control on cardiac output and peripheral vascular resistance, was similar between groups [50]. We did not detect any significant differences between groups in other measures of parasympathetically mediated autonomic control, including Valsalva and HRDB. While a significant difference in the maximum heart rate ratio was observed with Valsalva maneuver, the group mean was above a normal threshold and the difference did not persist in the per protocol analysis. The difference in heart rate variability on the LF/HF ratio with HUT was more likely to have been observed by chance when accounting for multiple comparisons. Finally, the data on sudomotor (sweat) response on QSART was insufficient to draw any conclusions.

Our findings may be consistent with experimental findings reported by other groups. In the mouse model of PWS (necdin-null mouse), loss of necdin impairs the survival and axonal elongation of sympathetic neurons and reduces the innervation of target glands with most rostral of sympathetic ganglia being affected [32]. Necdin appears to be crucial for the rostral migration and survival, but not proliferation, of the sympathetic superior cervical ganglion neurons; axonal extension is also impaired throughout the sympathetic nervous system. Impaired sympathetic function may explain, in part, the blunted heart rate response to HUT in the absence of evident parasympathetic dysfunction in PWS. Castner et al. also observed lower peak heart rate to a bicycle exercise test in PWS as compared with control weight and obese groups [51]. Additionally, in this study, all groups displayed similar heart rate recovery from peak exercise, while control (54 ± 16 beats) and obese group (50 ± 12 beats) subjects exhibited a significantly faster heart rate recovery from submaximal exercise than subjects with PWS (37 ± 14 beats) [51]. A delayed heart rate recovery in PWS from submaximal intensity exercise compared to normal weight controls also suggests possible ANS dysfunction. To the conclusion of the authors, these findings may be consistent with impaired sympathetically mediated heart rate responses to various stimuli in children with PWS. It should be noted that there are deletions of genes other than necdin in PWS; a major target is MAGEL2, which conveys information about autonomic status to the peripheral autonomic system [52]. Therefore, we might predict combined central and peripheral autonomic dysfunction in individuals with PWS.

The heart rate response to orthostatic stress like HUT reflects the combination of increased sympathetic and noradrenergic neurotransmission to the heart, and withdrawal of parasympathetic and vagal transmission. A blunted sympathetic and noradrenergic response in PWS compared with normal controls is one possible explanation of our findings given the absence of parasympathetic or vagally mediated dysfunction in other tests. However, all of the observed reflexes share sympathetic and parasympathetic inputs, and we did not perform specific tests of sympathetic function such as muscle sympathetic nerve activity (MSNA). Differences in peripheral vascular resistance and stroke volume may also contribute to group differences but were not measured.

Our findings also revealed no significant differences in LF and HF cardiac autonomic modulation or in the LF/HF ratio. These findings are in agreement with Baharav et al. who found no differences in cardiac autonomic modulation between patients with PWS and controls [53], and Wade et al., who found no difference in cardiac autonomic function during supine, standing, sitting, exercising or recovery measurements (LF, HF or LF:HF ratio) in 26 children with PWS compared to 26 age-, gender-, and BMI-matched controls [47]. Goldstone et al. reported abnormal (decreased) parasympathetic vagal innervation of the stomach as a possible reason for the elevation in ghrelin seen in PWS [54]. However, besides decreased salivation, we did not observe any evidence for parasympathetic dysfunction in PWS in the present study; interestingly, the necdin-null mice exhibit reduced sympathetic innervation of the intestinal tract but no defects in the parasympathetic innervation [32].

We observed notable differences in the pattern of symptoms reported by PWS and control participants on the ANS symptom questionnaire. Yet, decreased salivation and dry mouth were more frequently reported in PWS, as has been commonly observed [23,55,56]. The secretion of saliva is predominantly under parasympathetic control via the facial and glossopharyngeal cranial nerves [57], so decreased salivation may suggest alterations in parasympathetic control or innervation of the salivary glands.

Pain insensitivity is also a well-documented symptom in PWS. We did not observe any evidence of small fiber nerve dysfunction as assessed by the sudomotor (sweat) function test, but significant missing data make our observations problematic. Priano et al. conducted a detailed neurophysiological battery of tests in 14 adults with PWS, 10 controls with obesity but no diabetes, and 10 age-matched lean controls [21]. Normal electroneurographic, sympathetic skin responses, and somatosensory-evoked potentials were found in all three groups. Thermal and pain thresholds, but not vibration, were significantly elevated in PWS compared to obese or lean controls. They suggested that abnormalities of the small nociceptive neurons of the dorsal root ganglia or hypothalamic region may be implicated.

Other general neurological symptoms including lack of coordination, excessive daytime sleepiness, and fatigue were also commonly reported in PWS. Defects in gross motor performance may develop as a consequence of an abnormal body composition and decreased physical activity. However, growth hormone therapy has been shown to improve motor development and body composition in children with PWS [58,59].

Arousals and respiratory events (central and obstructive sleep apnea) disrupt sleep by producing prolonged awakenings and thus shortening total sleep time, which may cause daytime sleepiness in PWS. However, even when the quantity and quality of sleep appears sufficient, many children with PWS complain of excessive sleepiness and fatigue [60,61], which can be highly disruptive to the daily routines of patients and their families, and affects clinical care and learning [62]. Moreover, excessive sleepiness and circadian disruption can reduce energy expenditure and promote food-seeking behavior [63,64], thereby contributing to the overweight and obesity observed in PWS. The ANS plays key roles in appetite, food intake, hormone production, body temperature, and sleep [63]; thus deficits in ANS function in PWS may require interventions in order to forestall chronic disease. Some case reports suggested that modafinil may effectively treat the sleep disturbances and excessive daytime sleepiness in PWS, but more clinical trials are needed [65,66].

Individuals with PWS exhibited significantly higher levels of psychological dysfunction with multiple areas of disturbance compared to controls. Lack of emotional regulation, anxiety, and difficulties of social adaptation suggest disturbances in social and emotional competencies in these patients [67]. Current knowledge is incomplete, hindering appropriate clinical care; hence, more studies characterizing the emotional/behavioral functioning of people with PWS are required [67].

The unique constellation of symptoms observed in children with PWS based on an autonomic symptom questionnaire could be validated by some objective assessments. In our current study, the rise in heart rate during HUT testing was reduced in children with PWS compared to controls, suggesting possible altered ANS function in PWS and corroborates findings in other studies [50,51]. Given the difficulty and lack of standardized autonomic tests and well-established ranges of normal values for children, and non-availability of testing facilities nor experts [68], complete objective ANS testing could be used in future, to validate the autonomic symptom questionnaire as an alternative screener for detecting ANS function accurately in children with PWS.

Emerging evidence points to specific genetic causes explaining autonomic dysregulation in certain conditions [18,69]. In future, detailed molecular genetic testing might be carried out in order to isolate and identify the genes in question and make early personalized preventative and treatment plans [18,69].

A major strength of the current study is the recruitment and comparison of ANS function in a sizeable sample of children with PWS to controls. Given that PWS is a rare genetic condition, it is often difficult to recruit and administer a battery of tests on individuals who exhibit high levels of anxiety and other psychological and social disturbances.

The strengths of this study notwithstanding, we encountered some limitations as follows. The best standardized battery of tests presently available for testing ANS function were used in this study. However, there are known limitations in the testing of ANS function [68], especially in children. As such, children who participated in this study may have found it difficult adhering to the strict protocol for ANS testing provided in the lab. This may have resulted in some variability; for example, participants had difficulty breathing at the prescribed rate during the HRDB test and also had difficulty achieving and maintaining the required expiratory pressure during the Valsalva test, evidenced by the number of missing data points for each of these assessments (Table 2). Given that our focus, however, was the comparison of PWS and controls under the same conditions, the results are still valid across groups. Finally, we also observed missing data from both the PWS and control groups at random, further highlighting the difficulty associated with testing ANS function in children. Improvements to the comprehensiveness and protocol for testing ANS function in children are therefore needed to enhance reproducibility of tests and results in pediatric populations. Another limitation of the present study was the higher preponderance of female participants in the PWS group compared to controls. This precluded a systematic sex-stratified analysis. Despite more pronounced parasympathetic cardiac regulation, women have been reported to have a higher resting heart rate and lower baroreflex sensitivity [70]. Yet despite the predominance of females in the PWS group, their maximum heart rate responses were lower than those in the control group. Unfortunately, we did not investigate the origins of altered pain perception in participants with PWS.

## 4. Materials and Methods

### 4.1. Subjects

Children with genetically confirmed PWS (*n* = 20) were recruited for this study from pediatric endocrinology and genetics clinics at the University of Alberta, University of Calgary, and from across Canada and the United States. Age, weight z-score, and BMI z-score-matched controls (*n* = 27) were recruited from the Pediatric Centre for Weight and Health in Edmonton, Alberta. Potential participants were excluded if they had diabetes mellitus, chronic inflammatory bowel disease, severe liver or kidney disease, neurologic disorder, or claustrophobia. The study protocol was approved by the human research ethics committee of the University of Alberta (Pro00009903). All participants and parents provided written informed consent prior to taking part in the study.

### 4.2. Autonomic Symptom Questionnaire

An autonomic symptom questionnaire was used to assess symptoms of autonomic function in each of the following organ system domains: cardiovascular, respiratory, gastrointestinal and eating behaviours, urinary, temperature regulation, neurological, psychological, and vision [71,72,73,74]. Questions (e.g., “How often does your child experience difficulty emptying the bladder?”) were answered based on frequency of symptoms with the following ordinal options: never, <once per month, 2–4 times per month, 5–7 times per month, most days, and daily. Participants were encouraged to seek clarification if there was uncertainty about the question.

### 4.3. Pupillometry

Pupillometry, a test of sympathetic innervation of the pupil, was performed by applying a topical ophthalmologic solution of 5% cocaine to the conjunctiva of one eye of those participants who consented to the procedure [75,76]. After 15 min, the pupils were photographed without a flash and pupil diameter was measured. The online image processing software, ImageJ.JS 1.53m (https://ij.imjoy.io/ accessed on 10 January 2021), was used for analysis of pupil diameter.

### 4.4. Clinical Autonomic Cardiac Reflex Testing

A battery of standard ANS clinical assessments, including HRDB, Valsalva maneuver, 70 degrees HUT, and QSART were used to assess both cardiac autonomic reflexes and sudomotor function [71]. The electrocardiogram (ECG) was measured continuously with a digital sampling rate of 200 Hz and heart rate was derived from ECG waveform [77]. Finger arterial blood pressure was measured continuously during each procedure using a finger cuff and infrared plethysmograph (Finometer PRO, Finapress Medical Systems BV, Amsterdam, The Netherlands). All autonomic assessments and signals were captured in an integrated digital recording platform (WR TestWorks^®^, Stillwater, MN, USA). The HRDB procedure required the participant to follow a metronome to breathe at approximately 6 breaths per minute for a total of 8 breaths [71]. At least two attempts were made with a rest interval of 5 min in between. The average heart rate difference between inspiration and expiration for the best HRDB response was included in the analysis, and the time taken for 5 breaths was recorded. The Valsalva maneuver involved maintaining an expiratory pressure of 40 mm Hg for 15 s, blowing into a mouthpiece with high expiratory outflow resistance to increase intrathoracic pressure transiently [71]. The maximum heart rate during Valsalva and lower heart rate within 30 s after Vasalva was recorded as the heart ratio. The greatest heart rate ratio was included in the analysis from two Valsalva attempts conducted with a 5 min rest interval in between.

In addition, heart rate variability at rest and in response to HUT was assessed using time domain and frequency domain analysis [78]. For frequency domain analysis, the power density spectrum was integrated, and power spectra within the 0.04–0.15 Hz range were defined as LF components, whereas those at 0.15–0.4  Hz frequency were defined as HF components. Autoregressive spectral analysis was used to derive the frequency domain measures in high (HF), low (LF), and very low frequencies (VLF). The LF/HF power ratio was also determined as an indirect measure of sympathovagal balance [78].

QSART [79] was used to quantify postganglionic sweat output resulting from axon reflex stimulation using acetylcholine iontophoresis. The QSART device (Q-Sweat, WR Medical Inc., Stillwater, MN, USA) was used to measure sweat output, indicative of the sudomotor response at the foot, distal leg, proximal leg, and ipsilateral forearm.

### 4.5. Statistical Analysis

The a priori statistical analysis was performed on all available data. Considerable efforts were made to obtain high quality data from each participant, but the autonomic testing maneuvers described above were performed with variable degrees of adherence. As a quality and sensitivity analysis, a per protocol analysis was also performed where data from procedures with inadequate adherence to the protocol were excluded. Normally distributed continuous variables were represented as mean ± standard deviation (SD); group differences in these variables were compared by Student’s *t*-test. Other continuous variables were represented as medians and 25th and 75th percentiles of the interquartile ranges; group differences in these variables were compared by Wilcoxon’s rank test. Categorical variables were described as frequencies and percentages, with the between-group difference tested by means of the chi-square test or Fisher’s exact test when the number of events was less than 5. A *p*-value < 0.05 was considered statistically significant. The Statistical Package for the Social Sciences (SPSS) software (version 20.0; IBM Corp., Armonk, NY, USA) was used to analyze all data. R (version 4.2.2) [80], R-Studio (2022.12.0.353) [81], and tidyverse package [82] were used for the analysis of autonomic outcome measures, and the gtsummary package [83] was used for the formatting of reporting tables.

## 5. Conclusions

In summary, the increase of heart rate in response to HUT was reduced in children with PWS compared to controls, suggesting possible altered sympathetic function in PWS which is supported by the self- and parent-reported frequency of signs and symptoms associated with peripheral autonomic dysfunction compared to controls. There were no significant differences detected between groups for the other measures of parasympathetically mediated autonomic control, including Valsalva and HRDB. Our findings are supported by both studies in PWS mouse models and clinical trials of individuals with PWS. Future studies are needed to complete detailed molecular genetic testing to isolate and identify genes associated with central and peripheral autonomic dysfunction in PWS. Additionally, longitudinal studies of children and adults with PWS should determine if abnormalities in ANS function progress with age. These future studies may lead to the development of targeted treatment for ANS sympathetic dysfunction in PWS in order to improve exercise tolerance, gastrointestinal and respiratory function, and enhance overall quality of life. Future studies may also be used to validate the autonomic symptom questionnaire as a screening tool for detecting ANS dysfunction in individuals with PWS and enhance clinical care through early detection.

## Figures and Tables

**Table 1:** Characteristics of study participants.

	PWS			Controls			*p*-Values ^1^
	*n*	Median (25th, 75th Percentiles)	Mean (SD)	*n*	Median (25th, 75th Percentiles)	Mean (SD)	
Age, years	20	10.5 (7.1, 14.3)	10.8 (3.9)	27	12.8 (9.0, 14.0)	12.2 (3.1)	0.17
Male/Female	6/14	-	-	19/8	-	-	0.006 *
Weight, z-score	20	0.3 (−0.4, 1.0)	0.3 (1.3)	27	0.4 (−0.03, 0.9)	0.4 (0.9)	0.77
Height, z-score	20	−0.9 (−1.9, −0.2)	−0.9 (1.1)	27	0.2 (−0.4, 0.8)	0.2 (0.8)	0.0003 *
BMI, z-score	20	0.7 (0.2, 1.5)	0.8 (1.1)	27	0.2 (−0.5, 1.1)	0.3 (1.0)	0.15
WC (cm)	19	71.7 (56.5, 87.2)	76.1 (23.2)	24	67.0 (58.4, 72.4)	67.4 (15.8)	0.39

BMI z-scores were calculated using EpiInfo (CDC, Atlanta, GA). Independent samples *t*-test was used to evaluate differences between groups ^1^. Statistically significant (*p* < 0.05) *. Abbreviations: PWS, Prader–Willi syndrome; SD, standard deviation; BMI, body mass index; WC, waist circumference.

**Table 2:** Genetics of Prader–Willi syndrome participants.

Characteristic	Overall, *n* = 18 ^1^	Deletion, *n* = 12 ^1^	UPD, *n* = 6 ^1^	*p*-Value ^2^
Resting supine HR	74 (64, 82)	66 (60, 80)	81 (72, 85)	0.15
(Missing)	4	3	1	
Max HR	117 (111, 126)	117 (104, 126)	116 (113, 123)	>0.9
(Missing)	4	3	1	
Max change HR	41 (34, 47)	41 (36, 53)	38 (31, 42)	0.2
(Missing)	4	3	1	
Max change in SBP	−38 (−48, −28)	−42 (−55, −33)	−30 (−37, −28)	0.4
(Missing)	5	4	1	
Valsalva–HR ratio	1.61 (1.45, 1.91)	1.80 (1.62, 1.97)	1.50 (1.37, 1.63)	0.3
(Missing)	10	8	2	
Valsalva–HR ratio (per protocol)	1.91 (1.54, 1.92)	1.92 (1.68, 2.01)	1.73 (1.63, 1.82)	0.8
(Missing)	13	9	4	
HR Deep Breathing	22 (18, 28)	23 (19, 28)	20 (17, 21)	0.4
(Missing)	3	2	1	
HR Deep Breathing (per protocol)	22.4 (20.8, 26.1)	22.8 (21.1, 25.1)	21.9 (20.5, 29.8)	>0.9
(Missing)	8	5	3	
Duration of HRDB	45.0 (43.0, 45.0)	45.0 (37.5, 45.0)	45.0 (44.0, 45.5)	0.6
(Missing)	8	5	3	

Data presented as Median (Inter-Quartile Range (IQR) ^1^. Wilcoxon rank sum test and Wilcoxon rank sum exact test were used to evaluate differences between groups. *p*-value for independent groups *t*-test (approximately normal distribution); Mann–Whitney U test (non-normal distribution) ^2^. Statistically significant (*p* < 0.05). Abbreviations: UPD, uniparental disomy; HR, heart rate; SBP, systolic blood pressure, HRDB, heart rate deep breathing.

**Table 3:** Clinical Autonomic Cardiac Reflex Testing: Head-up tilt, Valsalva, Heart Rate Deep Breathing, and Quantitative Sudomotor Axon Reflex Test and group comparisons.

Characteristic	Group ^1^		*p*-Values ^2^
	Control *n* = 19	PWS *n* = 18	
Head-Up Tilt			
Resting supine HR			0.2
Median (IQR)	66 (61, 74)	74 (65, 82)	
(Missing)	1	2	
Max HR			0.12
Median (IQR)	124 (118, 132)	117 (112, 124)	
(Missing)	1	2	
Max change HR			0.002 *
Median (IQR)	55 (49, 60)	41 (35, 50)	
(Missing)	1	2	
Max change in SBP			0.4
Median (IQR)	−44 (−64, −34)	−38 (−47, −27)	
(Missing)	1	3	
Valsalva and Deep Breathing			
Valsalva—HR ratio			0.007 *
Median (IQR)	2.28 (1.94, 2.44)	1.68 (1.45, 1.92)	
(Missing)	1	9	
Valsalva—HR ratio (per protocol)			0.059
Median (IQR)	2.29 (2.00, 2.46)	1.92 (1.63, 2.06)	
(Missing)	2	12	
HR Deep Breathing			0.7
Median (IQR)	26 (20, 31)	22 (18, 29)	
(Missing)	1	1	
HR Deep Breathing (per protocol)			0.7
Median (IQR)	24 (18, 31)	23 (21, 27)	
(Missing)	3	7	
Duration of HRDB			0.12
Median (IQR)	45.0 (44.8, 46.2)	45.0 (42.5, 45.0)	
(Missing)	3	7	

Data presented as Median (Inter-Quartile Range (IQR) ^1^. *p*-value for independent groups *t*-test (approximately normal distribution); Mann–Whitney U test (non-normal distribution); Wilcoxon rank sum test and Wilcoxon rank sum exact test were used to evaluate differences between groups ^2^. Statistically significant (*p* < 0.05) *. Abbreviations: PWS, Prader–Willi syndrome; HR, Heart rate; SBP, systolic blood pressure; HRDB, heart rate deep breathing.

## Data Availability

The data are not publicly available due to ethical restrictions.
